# Resveratrol Protects against Physical Fatigue and Improves Exercise Performance in Mice

**DOI:** 10.3390/molecules18044689

**Published:** 2013-04-19

**Authors:** Ruei-Er Wu, Wen-Ching Huang, Chen-Chung Liao, Yu-Kai Chang, Nai-Wen Kan, Chi-Chang Huang

**Affiliations:** 1 Graduate Institute of Sports Science, College of Exercise and Health Sciences, National Taiwan Sport University, Taoyuan 33301, Taiwan; 2 Graduate Institute of Athletics and Coaching Science, College of Sports and Athletics, National Taiwan Sport University, Taoyuan 33301, Taiwan; 3 Proteomics Research Center, National Yang-Ming University, Taipei 11221, Taiwan; 4 Center for Liberal Arts, Taipei Medical University, Taipei 11031, Taiwan

**Keywords:** resveratrol, anti-fatigue, exercise performance, forelimb grip strength, ammonia, creatine kinase

## Abstract

Resveratrol (RES) is a well-known phytocompound and food component which has antioxidative and multifunctional bioactivities. However, there is limited evidence for the effects of RES on physical fatigue and exercise performance. The purpose of this study was to evaluate the potential beneficial effects of *trans*-RES on fatigue and ergogenic functions following physiological challenge. Male ICR mice from four groups (*n* = 8 per group) were orally administered RES for 21 days at 0, 25, 50, and 125 mg/kg/day, which were respectively designated the vehicle, RES-25, RES-50, and RES-125 groups. The anti-fatigue activity and exercise performance were evaluated using forelimb grip strength, exhaustive swimming time, and levels of serum lactate, ammonia, glucose, and creatine kinase (CK) after a 15-min swimming exercise. The exhaustive swimming time of the RES-25 group (24.72 ± 7.35 min) was significantly (*p* = 0.0179) longer than that of vehicle group (10.83 ± 1.15 min). A trend analysis revealed that RES treatments increased the grip strength. RES supplementation also produced dose-dependent decreases in serum lactate and ammonia levels and CK activity and also an increase in glucose levels in dose-dependent manners after the 15-min swimming test. The mechanism was related to the increased energy utilization (as blood glucose), and decreased serum levels of lactate, ammonia, and CK. Therefore, RES could be a potential agent with an anti-fatigue pharmacological effect.

## 1. Introduction

Resveratrol (RES, *trans*-3,4',5-trihydroxystilbene), which belongs to the stilbene class of polyphenolic compounds, is produced by plants in response to pathogen infection and a variety of stress conditions, such as climate alteration, and exposure to ozone, heavy metals, and sunlight [1,2]. RES is found in many plant species, including berries, grapes, peanuts, pines, * etc.* Fresh grape skins contain about 50~100 μg/g wet weight RES [3]. In plants, RES is mostly found in glycosylated forms and other minor conjugated forms that contain one or two methyl groups, a fatty acid, or a sulfate group. Glycosylation is known to protect a compound from degradation and also make it more stable, soluble, and easily absorbed from the gastrointestinal tract [4]. After oral administration, it can be metabolized by liver phase II drug-metabolizing enzymes into such water-soluble forms as RES-3-*O*-glucuronide and RES-3-*O*-sulfate. These metabolites are the predominant forms excreted in the urine and show a higher serum half-life compared to the parent compound, RES [5]. The bioavailability and efficacy of these RES metabolites are still unknown [6]. However, the absorption of RES is much more efficient by oral administration than other known polyphenols, such as quercetin and catechin [7].

In recent years, the various pharmacological activities of RES (antioxidative [8], anti-inflammatory [9], anti-cancer [10], anti-diabetic [11], anti-asthmatic [12] and antalgic [13] activities) were elucidated by *in vitro* and *in vivo* studies. In addition, the RES is known to increase energy utilization by potentiating mitochondrial function via activation of the SIRT1 signaling pathway. However, there are still relatively few studies that directly address the possible anti-fatigue function of RES. Fatigue is defined as physical and/or mental weariness resulting in negative impacts on exercise intensity, work performance, family life, and social relationships [14]. Physical fatigue can be accompanied by deterioration in functional performance [15]. There are a least two mechanisms that can explain the occurrence of physical fatigue: oxidative stress and energy exhaustion [16]. Exhaustive or intensive exercise can lead to the accumulation of excess reactive free radicals that result in tissue damage. Exhaustion theory suggests that energy source depletion and excess metabolite accumulation can lead to fatigue [17]. A previous article reviewed the physiological effects of RES on energy metabolism and utilization via SIRT1 activation [18]. Therefore, we conducted this study to evaluate the potential ergogenic and anti-fatigue effects of RES using our previously established *in vivo* platform [19,20]. 

## 2. Results and Discussion

### 2.1. Body Weight (BW), Skeletal Muscle Mass, and Other Metabolism-Related Organ Weights

**Figure 1:**
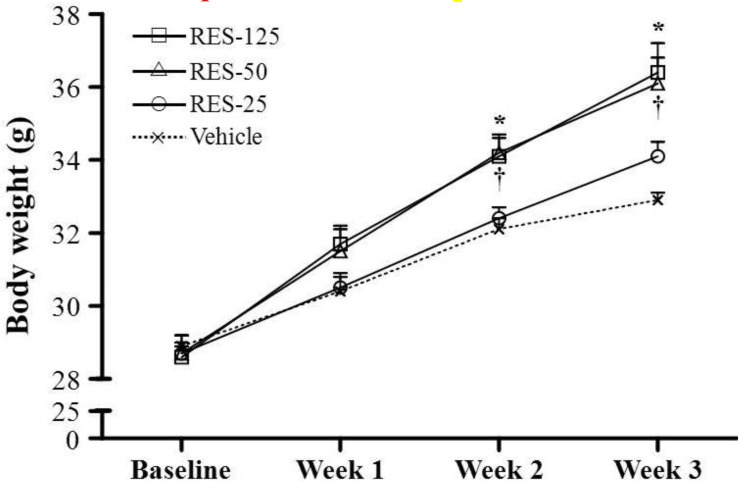
Increased body weight in resveratrol (RES)-treated mice. Male ICR mice were supplemented with vehicle, 25, 50 and 125 mg/kg resveratrol (RES-25, RES-50 and RES-125) for 21 days. Values are the mean ± SEM for n = 8 mice per group. *, *p* < 0.005, RES-125 *vs.* the vehicle control at weeks 2 and 3; **^†^**, *p* < 0.005, RES-50 *vs.* the vehicle control at weeks 2 and 3.

Morphological data from each experimental group are summarized in Table 1. There was no significant difference in initial BWs among the vehicle, RES-25, RES-50, and RES-125 groups. Because we observed a significant increase in cumulative food intake in RES-fed mice, the effects of RES on the BW and fat mass gain were of primary interest. The food intake and food efficiency ratio of the RES-50 and RES-125 groups were significantly higher by 1.40- (*p* = 0.0002) and 1.59-fold (*p* < 0.0001), respectively, compared to the vehicle group. Consistent with the food intake data in the RES-50 and RES-125 groups, we found the BWs of RES-50 and RES-125 groups were significant higher at weeks 2 and 3 (final BW) compared to the vehicle group (Figure 1). The trend analysis showed significant increases in the final BW (*p* < 0.0001), food intake (*p* < 0.0001), and food efficiency ratio (*p* = 0.0007) with an increasing dosage of RES supplementation. Therefore, the effect of RES on increasing the BW was clearly dependent on food intake. In addition, the trend analysis also showed significant increases in tissues weights of the liver (*p* = 0.0037), muscles (*p* = 0.0305), kidneys (*p* < 0.0001), and epididymal fat pads (EFPs) (*p* = 0.0482) with an increasing dosage of RES treatment. The relative tissue weight (%) is a measure of different tissue weights adjusted for the individual BW, and there were no significant changes in the relative liver, skeletal muscle (gastrocnemius and soleus muscles), kidney, or EFP weights (%) among the vehicle, RES-25, RES-50, and RES-125 groups.

**Table 1:** General characteristics of the experimental groups.

Characteristic	Vehicle	RES-25	RES-50	RES-125	Trend analysis
Initial BW (g)	28.9 ± 0.3	28.7 ± 0.5	28.7 ± 0.2	28.6 ± 0.4	0.4956
Final BW (g)	32.9 ± 0.2 ^a^	34.1 ± 0.4 ^a^	36.1 ± 0.7 ^b^	36.4 ± 0.8 ^b^	<0.0001
Food intake (g/day)	5.16 ± 0.14 ^a^	5.94 ± 0.12 ^a^	7.21 ± 0.30 ^b^	8.18 ± 0.50 ^c^	<0.0001
Food efficiency ratio	0.79 ± 0.02 ^a^	0.91 ± 0.02 ^ab^	1.03 ± 0.05 ^b^	0.97 ± 0.07 ^b^	0.0007
Liver (g)	1.82 ± 0.05 ^a^	1.84 ± 0.06 ^a^	1.94 ± 0.07 ^ab^	2.05 ± 0.05 ^b^	0.0037
Muscle (g)	0.33 ± 0.01 ^a^	0.34 ± 0.01 ^a^	0.38 ± 0.01 ^b^	0.36 ± 0.01 ^ab^	0.0305
Kidney (g)	0.52 ± 0.01 ^a^	0.59 ± 0.02 ^ab^	0.62 ± 0.03 ^b^	0.65 ± 0.01 ^b^	<0.0001
EFP (g)	0.54 ± 0.02 ^ab^	0.51 ± 0.02 ^a^	0.62 ± 0.04 ^ab^	0.69 ± 0.07 ^b^	0.0482
Relative liver weight (%)	5.57 ± 0.16	5.49 ± 0.12	5.37 ± 0.11	5.64 ± 0.18	0.8797
Relative muscle weight (%)	1.01 ± 0.03	1.01 ± 0.03	1.06 ± 0.03	0.99 ± 0.03	0.9878
Relative kidney weight (%)	1.59 ± 0.04 ^a^	1.76 ± 0.06 ^ab^	1.70 ± 0.06 ^ab^	1.79 ± 0.05 ^b^	0.0887
Relative EFP weight (%)	1.65 ± 0.08	1.51 ± 0.08	1.71 ± 0.11	1.87 ± 0.18	0.3194

Values are the mean ± SEM for *n* = 8 mice in each group. Values in the same line with different superscripts letters (a, b, c) differ significantly, *p* < 0.05 by one-way ANOVA. Food efficiency ratio: BW gain (g/day)/food intake (g/day). Muscle mass includes both gastrocnemius and soleus muscles in the back part of the lower legs. EFP: epididymal fat pad.

### 2.2. Effect of RES Supplementation on Forelimb Grip Strength

**Figure 2:**
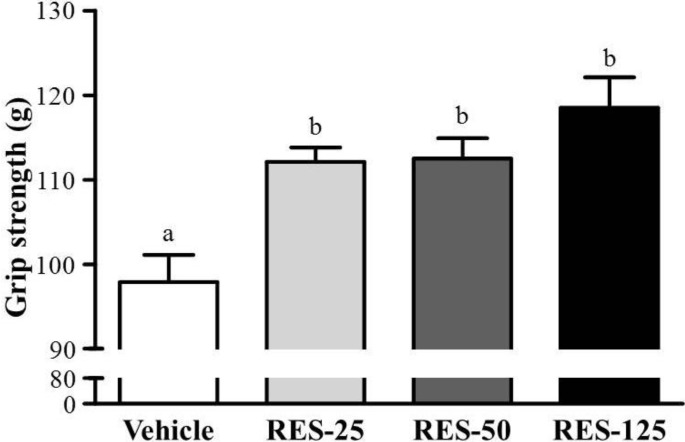
Effect of resveratrol(RES) supplementation on forelimb grip strength. Male ICR mice were pretreated with the vehicle, 25 (RES-25), 50 (RES-50), and 125 mg/kg RES (RES-125) for 21 days, and underwent a grip strength test 1 h after the final administered dose. Data are presentedasthe mean ± SEM of eight mice in each group. One-way ANOVA was used for the analysis. Different letters(a, b) indicate a significant difference at p < 0.05.

As shown in the Figure 2, respective data for grip strength were 97.9 ± 3.2, 112.1 ± 1.7, 112.5 ± 2.4, and 118.5 ± 3.6 g in the vehicle, RES-25, RES-50, and RES-125 groups, which were significantly higher by 1.15- (*p* = 0.0109), 1.15- (*p* = 0.0092) and 1.21-fold (*p* < 0.0005), respectively, compared to the vehicle control group. In the trend analysis, grip strength dose-dependently increased with the RES dose (*p* = 0.0013). A regulatory training program is needed for grip strength elevation, and results showed that RES treatment benefited grip strength without training intervention conditions. This result suggests that RES can improve the grip strength under a programmed training protocol.

### 2.3. Effect of RES Supplementation on Exercise Performance in a Weight-loaded Swimming Test

**Figure 3:**
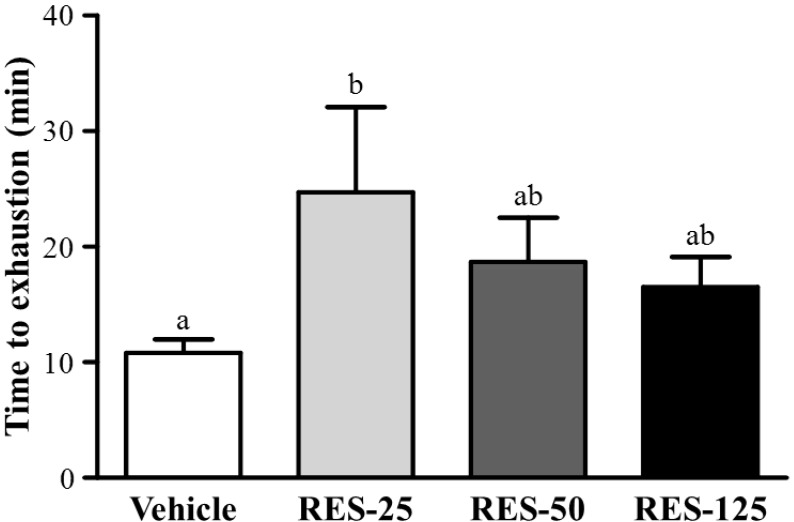
Effect of resveratrol (RES) supplementation on swimming exercise performance. Mice were pretreated with the vehicle, 25, 50, and 125 mg/kg of RES for 21 days and, then 1 h later performed an exhaustive swimming exercise with a load equivalent to 5% of the mouse’s bodyweight attached to its tail. Data represent the mean ± SEM (*n* = 8 mice). One-way ANOVA was used for the analysis. Different letters (a, b) indicate a significant difference at p < 0.05.

Energy metabolism during muscular activity determines the level of physiological fatigue [21]. Exercise endurance is an important variable in evaluating anti-fatigue treatment. In our study, the exercise endurance levels with a swimming test in mice administered the vehicle, RES-25, RES-50, and RES-125 were 10.83 ± 1.15, 24.72 ± 7.35, 18.68 ± 3.84, and 16.50 ± 2.60 min, respectively, as shown in Figure 3. The swimming time was significantly longer by 2.28-fold (*p* = 0.0179) with RES-25 compared to vehicle treatment. However, there were no significant differences in swimming times among the vehicle, RES-50, and RES-125 groups. This result conformed to a previous cognitive study with RES supplementation. At the higher RES doses, results also showed that exercise performance did not significantly increase but the cognitive performance was significantly elevated with RES supplementation [22]*.* These data indicate that selective concentrations of RES may differently contribute to physiological activities, and a 25 mg/kg dose may be the optimal range for endurance capacity.

### 2.4. Effect of RES Supplementation on Serum Lactate, Ammonia, Glucose, and CK Levels after Acute Exercise Challenge

**Figure 4:**
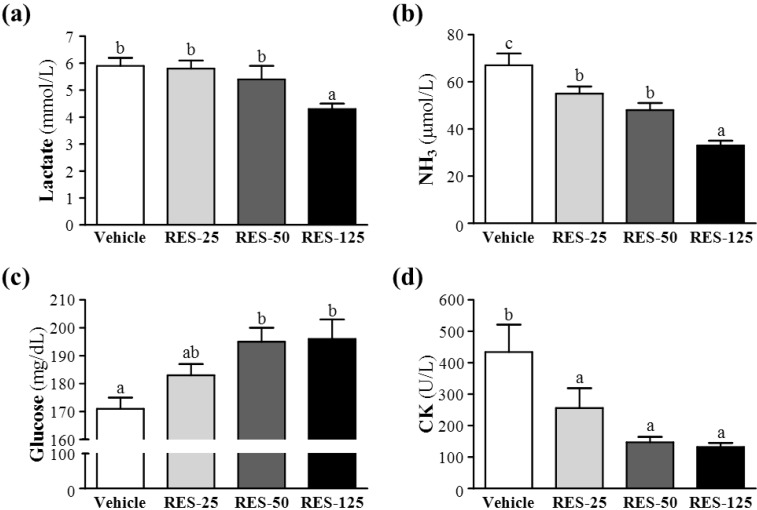
Effects of resveratrol(RES) supplementation on serum (**a**) lactate, (**b**) ammonia, (**c**) glucose, and (**d**) creatine kinase (CK) levels after an acute exercise challenge.Mice were pretreated with the vehicle, 25, 50, and 125 mg/kg of RES for 21 days, then 1 h later performed a 15-min swimming test without weight-loading. Data represent the mean ± SEMof eight mice in each group. Columns with different letters (a, b) significantly differ, *p* < 0.05 by a one-way ANOVA.

Muscle fatigue after exercise can be evaluated by important biochemical indicators, including lactate, ammonia, glucose, and CK, after exercise [23]. During high-intensity exercise, muscles must obtain sufficient energy from anaerobic glycolysis, and abundant lactate is produced by the process of glycolysis metabolism. The increased lactate level further reduces the pH value, which can result in various biochemical and physiological side effects on glycolysis, phosphofructokinase, and muscular contractions caused by calcium ion release [24]. In the present study, respective lactate levels in the vehicle, RES-25, RES-50, and RES-125 groups were 5.9 ± 0.3, 5.8 ± 0.3, 5.4 ± 0.5, and 4.3 ± 0.2 mmol/L; only RES-125 treatment was significantly lower by 27.14% (*p* = 0.0023) than the vehicle treatment (Figure 4a).

Ammonia, a metabolite of proteins and amino acids, was linked to fatigue as early as 1922. The immediate source of ammonia produced from deamination of AMP to inosine monophosphate (IMP) via the purine nucleotide cycle is the greatest during intensive exercise when the rate of ATP utilization may exceed the rate of ATP production. Muscle fatigue is associated with deamination of adenine nucleotides, and increased deamination of AMP coincides with decreases in phosphocreatine and pH values, and failure of the contraction process. Peripheral and central fatigue levels are related to increased ammonia during exercise [25]. Serum ammonia levels in the vehicle, RES-25, RES-50, and RES-125 groups were 67 ± 5, 55 ± 3, 48 ± 3, and 33 ± 2 μmol/L, respectively. Values of the RES-25, RES-50, and RES-125 groups were significantly lower, by 18% (*p* = 0.0168), 28% (*p* = 0.0004), and 50% (*p* < 0.0001), respectively, than that of the vehicle control (Figure 4b).

Glucose, a breakdown product of tissue glycogen, is released as a circulating substrate for energy utilization after intense exercise [26]. During exercise, the plasma glucose is increased by the combined actions of glucagon, epinephrine, norepinephrine, and cortisol. Although the insulin helps glucose enter the body cell, but it declines during prolonged exercise. Exercise and muscle contractions increase glucose uptake by skeletal muscles by a mechanism that is independent of the insulin signaling pathway [27]. Glucose uptake significantly increased by 26% due to muscle contractions [28]. Therefore, blood glucose levels are an important index for performance maintenance during exercise. Respective levels of serum glucose in the vehicle, RES-25, RES-50, and RES-125 groups were 171 ± 4, 183 ± 4, 195 ± 5, and 196 ± 7 mg/dL, and those of the RES-50 and RES-125 groups were respectively significantly higher, by 1.14- (*p* = 0.0028) and 1.15-fold (*p* = 0.0015), compared to that of the vehicle control (Figure 4c).

Excessive oxidative free radicals produced by intensive exercise cannot be coped with by the physiological antioxidant defense system and result in lipid peroxidation which destroys the cell organization, and DNA integrity and function. Under oxidative stress-induced cellular injuries, the cell membrane integrity can be damaged, and cytosolic enzymes will leak out into the serum. Those enzymes, including lactate dehydrogenase (LDH), CK, myoglobin, aspartate aminotransferase (AST), alanine aminotransferase (ALT), *etc.*, can be parameters indicating tissue injury under high-intensity exercise challenge. Serum CK is an important clinical biomarker for muscle damage, such as muscular dystrophy, severe muscle breakdown, myocardial infarction, autoimmune myositides, and acute renal failure. CK activities in the vehicle, RES-25, RES-50, and RES-125 groups were 434 ± 87, 256 ± 63, 147 ± 17 and 132 ± 13 U/L, respectively (Figure 3d), and those of the RES-25, RES-50, and RES-125 groups were significantly lower, by 41% (*p* = 0.0295), 66% (*p* = 0.0009), and 70% (*p* = 0.0006), respectively, than that of the vehicle control (Figure 4d). Therefore, RES supplementation should ameliorate skeletal muscle injury induced by acute exercise challenge. The trend analysis revealed that RES treatment had a significant dose-dependent effect on blood glucose levels (*p* < 0.0001) and dose-dependently decreased serum lactate and ammonia levels and CK activity at *p* < 0.0001.

### 2.5. Effect of RES Supplementation on Hepatic Glycogen Level

**Figure 5:**
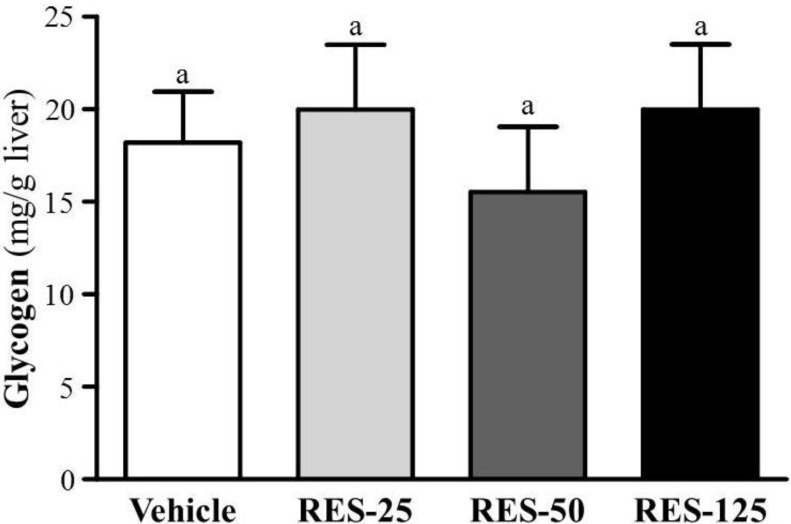
Effect of resveratrol (RES) on hepatic glycogen levels at rest. Mice were pretreated with the vehicle, and 25, 50, and 125 mg/kg of RES for 21 days. All mice were sacrificed and examined for glycogen levels of muscle and liver tissues 1 h after the final treatment. Data represent the mean ± SEM of eight mice in each group.

With energy expenditure during exercise, physical fatigue is mainly caused by energy consumption and deficiency [29]. Catabolized fat and carbohydrates are considered the main sources of energy during exercise in skeletal muscles, and glycogen is the predominant source of glycolysis for energy production. Therefore, glycogen storage directly affects exercise ability [30]. RES, fasting, and calorie restriction (CR) can activate the expression and biological activity of SIRT1 which plays a key role in regulating mitochondrial biogenesis and energy metabolism in different related tissues such as the liver, skeletal muscles, adipose tissues, and the pancreas [18]. PGC-1α activation by SIRT1 in liver cells can result in downregulation of glycolytic pathways and upregulation of gluconeogenic pathways [31]. Therefore, we found the glycogen contents of liver tissues did not show significant differences among groups in our data (Figure 5). In a previous study, SIRT1 also directly activated FOXO1 to shift glucose metabolism to gluconeogenesis, and promoted the release of glucose [32]. As shown in our data, that could explain the significant dose-dependent increased glucose levels from the trend analysis (*p* < 0.0001) in Figure 4c.

### 2.6. Effect of RES Supplementation on Biochemical Analyses at the End of the Experiment

**Figure 6:**
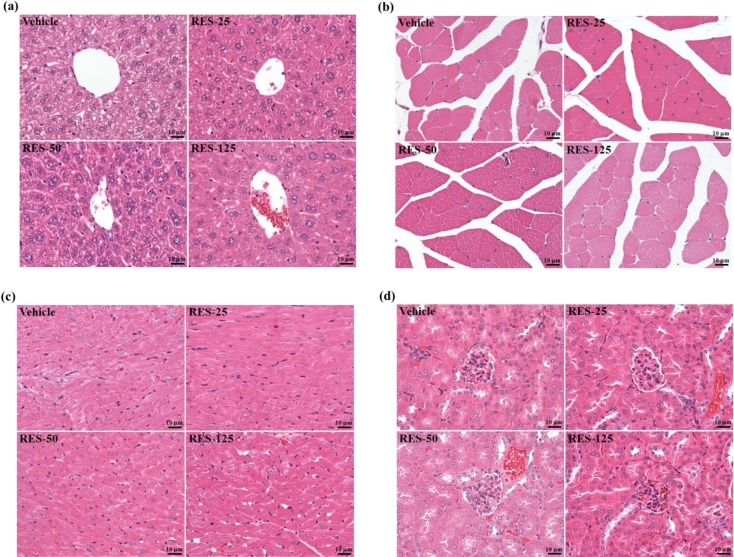
Effect of resveratrol supplementation on the morphology of liver (**a**), skeletal muscle (**b**), heart (**c**), and kidney (**d**) tissues. Specimens were photographed with a light microscope (Olympus BX51). (H&E stain, magnification: ×400, Scale bar, 10 μm).

In the present study, we observed beneficial effects of RES on the exhaustive exercise challenge and measured other physiological effects with 3 weeks of RES supplementation. In the Table 2, we found the serum AST level in the RES-125 group significantly decreased by 23.4% (*p = *0.0104), and the serum LDH levels in the RES-50 and RES-125 groups were significantly lower than that of the vehicle control group by 21.6% (*p =* 0.05) and 38.4% (*p =* 0.001), respectively. A previous study reported that RES provided a protective effect against chemical-induced oxidative stress on AST, ALT, and LDH activities, and creatinine and blood urea nitrogen (BUN) levels [33]. As to kidney function, the biochemistry of urea, creatinine, and uric acid (UA) can reflect renal damage. During renal ischemia-reperfusion (I/R) injury, the burst of reactive oxygen species (ROS), mainly produced by xanthine oxidase, can trigger inflammation and tubular cell injury and subsequent generation of UA [34]. The current data of kidney-related parameters showed that creatinine significantly decreased in the RES-125 group by 7.1% (*p =* 0.03) and UA in the RES-50 and RES-125 groups were significantly lower by 21.6% (*p =* 0.05) and 38.4% (*p =* 0.001), respectively, compared to values of the vehicle control group. Therefore, RES may have potential for renal protection due to its antioxidant activity. Our results also showed that RES supplementation had no toxic effects on major organs such as the liver, skeletal muscles, heart, or kidney according to histopathological examinations (Figure 6).

Mice on a high-fat diet exhibited increase mitochondrial activity/content in brown adipose tissues, skeletal muscles, and the liver, while RES supplementation protected against diet-induced obesity and metabolic disturbances [35]. Recent animal studies demonstrated a promising perspective to ameliorate serious metabolic disorders such as obesity with the potential use of RES [36], and its mechanism could possibly be downregulation of peroxisome proliferator-activated receptor gamma (PPARγ) and C/EBPα gene expressions followed by suppression of adipogenesis genes that regulate lipid synthesis and accumulation [37]. With continuous RES supplementation without a high-fat diet, triglycerides (TGs) significantly decreased by about 36.8% (*p =* 0.0017 and *p =* 0.002, respectively) in the RES-50 and RES-125 groups compared to the vehicle group. Above all, RES may possibly have potential applications for liver and renal protection and also for hyperlipidemia according to our *in vivo* data.

**Table 2:** Biochemical analysis of the resveratrol (RES) treatment groupsat the end of the experiment.

Parameter	Vehicle	RES-25	RES-50	RES-125	Trend analysis
AST (U/L)	64 ± 4 ^b^	61 ± 4 ^b^	62 ± 3 ^b^	49 ± 1 ^a^	0.0036
ALT (U/L)	32 ± 2	33 ± 2	38 ± 3	31 ± 1	0.9216
ALP (U/L)	287 ± 28	315 ± 11	284 ± 15	292 ± 10	0.7657
LDH (U/L)	416 ± 27 ^c^	388 ± 39 ^bc^	326 ± 29 ^ab^	256 ± 5 ^a^	<0.0001
Albumin (g/dL)	3.3 ± 0.0	3.3 ± 0.0	3.3 ± 0.1	3.2 ± 0.0	0.2861
TBIL (μg/dL)	95 ± 7	84 ± 5	96 ± 5	99 ± 6	0.6861
TP (g/dL)	5.6 ± 0.1	5.6 ± 0.1	5.5 ± 0.1	5.5 ± 0.0	0.1361
BUN (mg/dL)	21.0 ± 0.6	19.3 ± 0.5	20.9 ± 0.8	20.0 ± 0.5	0.7720
Creatinine (mg/dL)	0.28 ± 0.01 ^b^	0.28 ± 0.01 ^ab^	0.27 ± 0.01 ^ab^	0.26 ± 0.01 ^a^	0.0087
UA (mg/dL)	1.94 ± 0.14 ^c^	1.66 ± 0.11 ^bc^	1.20 ± 0.22 ^ab^	0.80 ± 0.07 ^a^	<0.0001
TC (mg/dL)	148 ± 4	144 ± 4	145 ± 5	156 ± 4	0.2133
TG (mg/dL)	103 ± 10 ^b^	83 ± 7 ^ab^	65 ± 6 ^ a^	66 ± 3 ^a^	<0.0001
Glucose (mg/dL)	191 ± 6	189 ± 4	178 ± 4	195 ± 4	0.8109

Values are mean ± SEM for n = 8 mice per group. Values in the same line with different superscripts letters (a, b, c) differ significantly, *p* < 0.05 by one-way ANOVA. AST, aspartate aminotransferase; ALT, alanine aminotransferase; ALP, alkaline phosphatase; LDH, lactate dehydrogenase; TBIL, total bilirubin; TP, total protein; BUN, blood urea nitrogen; UA, uric acid; TC, total cholesterol; TG, triacylglycerol.

## 3. Experimental

### 3.1. Materials, Animals, and Experiment Design

Trans-RES used for supplementation in the study was purchased from Biotivia LLC (Fenix Biotechnology Ltd., New Taipei City, Taiwan). Male ICR strain mice (6 weeks old) with specific pathogen free (SPF) conditions were purchased from BioLASCO (A Charles River Licensee Corp., Yi-Lan, Taiwan). One week of acclimation to the environment and diet was allowed before the experiment began. All animals were provided with a standard laboratory diet (No. 5001; PMI Nutrition International, Brentwood, MO, USA) and distilled water *ad libitum*, and housed at 12-h light/12-h dark cycle at room temperature (24 ± 1 °C) and 50%~60% humidity. The Institutional Animal Care and Use Committee (IACUC) of National Taiwan Sport University (NTSU) inspected all animal experiments in this study, and this study conformed to the guidelines of protocol IACUC-10109 approved by the IACUC ethics committee. All animals were randomly assigned to four groups (eight mice/group) for RES treatment: (1) vehicle; (2) 25 mg/kg RES (RES-25); (3) 50 mg/kg RES (RES-50); and (4) 125 mg/kg RES (RES-125). The same volume of solution equivalent to individual BWs was received by the vehicle group. Both the vehicle and RES in this experiment were administrated by oral gavage.

### 3.2. Forelimb Grip Strength

A low-force testing system (Model-RX-5, Aikoh Engineering, Nagoya, Japan) was used to measure the forelimb grip strength of mice undergoing vehicle or RES treatments. The amount of tensile force was measured by a force transducer equipped with a mental bar (2 mm in diameter and 7.5 cm in length) for each mouse in the different groups. The detailed procedures were described in our previous studies [19,20]. The test of forelimb grip strength was performed after consecutive administration of the vehicle of RES for 21 days and 1 hour after the last treatment. The maximal force (in grams) recorded by this low-force system was used as the grip strength.

### 3.3. Swimming Exercise Performance Test

Mice were pretreated with the vehicle, or 25, 50, or 125 mg RES/kg for 21 continuous days, followed by an exhaustive swimming test which began 1 h after the last administration. Details of the swimming exercise performance in the forced swimming test were as previously described [19,20] to evaluate endurance. The endurance of each mouse was recorded as the time from the beginning to exhaustion, which was determined by observing loss of coordinated movements and failure to return to the surface within 7 s. Times floating, struggling, and making necessary movements were considered in the swimming duration until exhaustion and possible drowning.

### 3.4. Determination of Blood Biochemical Variables

The effects of RES on serum lactate, ammonia, and glucose levels, and CK activity were evaluated post-exercise. One hour after the last administration, a 15-min swimming test was performed without weight-loading. After the swimming exercise, blood samples were immediately collected from the submandibular duct of pretreated mice and centrifuged at 1,500 ×*g* and 4 °C for 10 min for serum preparation. Lactate, ammonia, and glucose levels and CK activity in the serum were determined using an autoanalyzer (Hitachi 7060, Hitachi, Tokyo, Japan). The other biochemical variables, as shown in Table 2, were measured using an autoanalyzer (Hitachi 7080) after 3 weeks of RES supplementation without exercise.

### 3.5. Tissue Glycogen Determination

Liver tissues were investigated to see whether RES administration increased the content of glycogen deposition. Mice were continuously pretreated with the vehicle, or 25, 50, and 125 mg RES/kg for 21 days, and then sacrificed 1 h after the last treatment administration. The liver was excised and weighed for a subsequent glycogen content analysis. The method of glycogen analysis was described in our previous studies [19,20].

### 3.6. Histological Staining of Tissues

Different tissues were collected and fixed in 10% formalin after sacrifice. They were cut transversely or longitudinally to obtain ventricular sections or four-chamber cross-sections, respectively. Tissues were then embedded in paraffin and cut into 4-μm thick slices for morphological and pathological evaluations. Tissue sections were stained with hematoxylin and eosin (H&E) and examined using a light microscope equipped with a CCD camera (BX-51, Olympus, Tokyo, Japan) by a clinical pathologist.

### 3.7. Statistical Analysis

All data are expressed as the mean ± SEM. Statistical differences among groups were analyzed by a one-way analysis of variance (ANOVA) and the Cochran-Armitage test for the dose-effect trend analysis with SAS ver. 9.0 (SAS Institute, Cary, NC, USA). *p* values of <0.05 were considered statistically significant.

## 4. Conclusions

In this study, we found that 3-week RES supplementation significantly increased the body weight gain, food intake, and food efficiency ratio in the RES-50 and RES-125 groups, and showed beneficial effects on the lipid profile, and liver and renal functions. The exercise performance significantly increased in the RES-25 group. In addition, exercise-induced fatigue-related parameters, including lactate, ammonia, glucose, and CK, were positively modulated by RES supplementation in a dosage-dependent manner (trend analysis, *p* < 0.0001). Therefore, we suggest that RES may be a potential ergogenic aid against abnormal metabolite accumulation and to increase utilization of important fuel source (glucose).

## References

[1] Delmas D., Lancon A., Colin D., Jannin B., Latruffe N. (2006). Resveratrol as a chemopreventive agent: A promising molecule for fighting cancer. Curr. Drug Targets.

[2] Bavaresco L. (2003). Role of viticultural factors on stilbene concentrations of grapes and wine. Drugs Exp. Clin. Res..

[3] Joe A.K., Liu H., Suzui M., Vural M.E., Xiao D., Weinstein I.B. (2002). Resveratrol induces growth inhibition, S-phase arrest, apoptosis, and changes in biomarker expression in several human cancer cell lines. Clin. Cancer Res..

[4] Regev-Shoshani G., Shoseyov O., Bilkis I., Kerem Z. (2003). Glycosylation of resveratrol protects it from enzymic oxidation. Biochem. J..

[5] Walle T., Hsieh F., DeLegge M.H., Oatis J.E., Walle U.K. (2004). High absorption buy very low bioavailability of oral resveratrol in humans. Drug Metab. Dispos..

[6] Baur J.A., Sinclair D.A. (2006). Therapeutic potential of resveratrol: the *in vivo* evidence. Nat. Rev. Drug Discov..

[7] Soleas G.J., Yan J., Goldberg D.M. (2001). Ultrasensitive assay for three polyphenols (catechin, quercetin and resveratrol) and their conjugates in biological fluids utilizing gas chromatography with mass selective detection. J. Chromatogr. B Biomed. Sci. Appl..

[8] Zhao H., Niu Q., Li X., Liu T., Xu Y., Han H., Wang W., Fan N., Tian Q., Zhang H., Wang Z. (2012). Long-term resveratrol consumption protects ovariectomized rats chronically treated with D-galactose from developing memory decline without effects on the uterus. Brain Res..

[9] Busch F., Mobasheri A., Shayan P., Lueders C., Stahlmann R., Shakibaei M. (2012). Resveratrol modulates interleukin-1β-induced phosphatidylinositol 3-kinase and nuclear factor κB signaling pathways in human tenocytes. J. Biol. Chem..

[10] Castillo-Pichardo L., Dharmawardhane S.F. (2012). Grape polyphenols inhibit Akt/mammalian target of rapamycin signaling and potentiate the effects of gefitinib in breast cancer. Nutr. Cancer.

[11] Ramar M., Manikandan B., Raman T., Priyadarsini A., Palanisamy S., Velayudam M., Munusamy A., Marimuthu Prabhu N., Vaseeharan B. (2012). Protective effect of ferulic acid and resveratrol against alloxan-induced diabetes in mice. Eur. J. Pharmacol..

[12] Ichikawa T., Hayashi R., Suzuki K., Imanishi S., Kambara K., Okazawa S., Inomata M., Yamada T., Yamazaki Y., Koshimizu Y. (2013). The Sirt1 activator SRT1720 suppresses inflammation in an ova-induced mouse model of asthma. Respirology.

[13] Falchi M., Bertelli A., Galazzo R., Viganò P., Dib B. (2010). Central antalgic activity of resveratrol. Arch. Ital. Biol..

[14] Mehta R.K., Agnew M.J. (2012). Influence of mental workload on muscle endurance, fatigue, and recovery during intermittent static work. Eur. J. Appl. Physiol..

[15] Fitts R.H. (1994). Cellular mechanisms of muscle fatigue. Physiol. Rev..

[16] Coombes J.S., Rowell B., Dodd S.L., Demirel H.A., Naito H., Powers S.K. (2002). Effects of vitamin E deficiency on fatigue and muscle contractile properties. Eur. J. Appl. Physiol..

[17] You L., Zhao M., Regenstein J.M., Ren J. (2011). *In vitro* antioxidant activity and *in vivo* anti-fatigue effect of loach (*Misgurnus anguillicaudatus*) peptides prepared by papain digestion. Food Chem..

[18] Schwer B., Verdin E. (2008). Conserved metabolic regulatory functions of sirtuins. Cell Metab..

[19] Huang C.C., Hsu M.C., Huang W.C., Yang H.R., Hou C.C. (2012). Triterpenoid-rich extract from *Antrodia camphorata* improves physical fatigue and exercise performance in mice. Evid. Based Complement. Alternat. Med..

[20] Wang S.Y., Huang W.C., Liu C.C., Wang M.F., Ho C.S., Huang W.P., Hou C.C., Chuang H.L., Huang C.C. (2012). Pumpkin (*Cucurbita moschata*) fruit extract improves physical fatigue and exercise performance in mice. Molecules.

[21] Belluardo N., Westerblad H., Mudó G., Casabona A., Bruton J., Caniglia G., Pastoris O., Grassi F., Ibáñez C.F. (2001). Neuromuscular junction disassembly and muscle fatigue in mice lacking neurotrophin-4. Mol. Cell. Neurosci..

[22] Dal-Pan A., Pifferi F., Marchal J., Picq J.L., Aujard F. (2011). RESTRIKAL Consortium. Cognitive performances are selectively enhanced during chronic caloric restriction or resveratrol supplementation in a primate. PLoS One.

[23] Brancaccio P., Maffulli N., Limongelli F.M. (2007). Creatine kinase monitoring in sport medicine. Br. Med. Bull..

[24] Cairns S.P. (2006). Lactic acid and exercise performance: Culprit or friend?. Sports Med..

[25] Carvalho-Peixoto J., Alves R.C., Cameron L.C. (2007). Glutamine and carbohydrate supplements reduce ammonemia increase during endurance field exercise. Appl. Physiol. Nutr. Metab..

[26] Suh S.H., Paik I.Y., Jacobs K. (2007). Regulation of blood glucose homeostasis during prolonged exercise. Mol. Cells.

[27] Fujii N., Jessen N., Goodyear L.J. (2006). AMP-activated protein kinase and the regulation of glucose transport. Am. J. Physiol. Endocrinol. Metab..

[28] Manabe Y., Miyatake S., Takagi M., Nakamura M., Okeda A., Nakano T., Hirshman M.F., Goodyear L.J., Fujii N.L. (2012). Characterization of an acute muscle contraction model using cultured C2C12 myotubes. PLoS One.

[29] Sahlin K., Tonkonogi M., Söderlund K. (1998). Energy supply and muscle fatigue in humans. Acta Physiol. Scand..

[30] Young A.J., Castellani J.W. (2001). Exertion-induced fatigue and thermoregulation in the cold. Comp. Biochem. Physiol. A Mol. Integr. Physiol..

[31] Rodgers J.T., Lerin C., Haas W., Gygi S.P., Spiegelman B.M., Puigserver P. (2005). Nutrient control of glucose homeostasis through a complex of PGC-1alpha and SIRT1. Nature.

[32] Frescas D., Valenti L., Accili D. (2005). Nuclear trapping of the forkhead transcription factor FoxO1 via Sirt-dependent deacetylation promotes expression of glucogenetic genes. J. Biol. Chem..

[33] Sehirli O., Tozan A., Omurtag G.Z., Cetinel S., Contuk G., Gedik N., Sener G. (2008). Protective effect of resveratrol against naphthalene-induced oxidative stress in mice. Ecotoxicol. Environ. Saf..

[34] Tsuda H., Kawada N., Kaimori J.Y., Kitamura H., Moriyama T., Rakugi H., Takahara S., Isaka Y. (2012). Febuxostat suppressed renal ischemia-reperfusion injury via reduced oxidative stress. Biochem. Biophys. Res. Commun..

[35] Lagouge M., Argmann C., Gerhart-Hines Z., Meziane H., Lerin C., Daussin F., Messadeq N., Milne J., Lambert P., Elliott P. (2006). Resveratrol improves mitochondrial function and protects against metabolic disease by activating SIRT1 and PGC-1α. Cell.

[36] Szkudelska K., Szkudelski T. (2010). Resveratrol, obesity and diabetes. Eur. J. Pharmacol..

[37] Zhang X.H., Huang B., Choi S.K., Seo J.S. (2012). Anti-obesity effect of resveratrol-amplified grape skin extracts on 3T3-L1 adipocytes differentiation. Nutr. Res. Pract..

